# Nutraceutical Impact of Pumpkin Seed Oil on Expression Levels of EZH-2 and KRT-14 Genes against DSS-induced Inflammatory Bowel Disease in the Rat Model

**DOI:** 10.1007/s12013-024-01629-7

**Published:** 2025-01-27

**Authors:** Asma Mukhtar, Imran Mukhtar, Humaira Muzaffar, Muhammad Naeem Faisal, Kashif ur rehman khan, Laaraib Nawaz, Muhammad Umar Ijaz, Sana Maryam, Muhammad Umair, Haseeb Anwar, Farid S. Ataya, Gaber El-Saber Batiha, Athanasios Alexiou, Marios Papadakis, Nermeen N. Welson, Sameh A. Korma

**Affiliations:** 1 Health Biology Lab, Department of Physiology, Government College University, Faisalabad, Pakistan; 2 Institue of Physiology and Pharmacology, University of Agriculture, Faisalabad, Pakistan; 3 Department of Pharmaceutical Chemistry, Faculty of Pharmacy, The Islamia University Bahawalpur, Bahawalpur, Pakistan; 4 Department of Zoology, Wildlife and Fisheries, University of Agriculture, Faisalabad, Pakistan; 5 Department of Pharmacology, Faculty of Pharmacy, The Islamia University Bahawalpur, Bahawalpur, Pakistan; 6 College of Pharmacy, Shenzhen Technology University, Shenzhen, China; 7 Department of Biochemistry, College of Science, King Saud University, PO Box 2455, Riyadh, 11451 Saudi Arabia; 8 Department of Pharmacology and Therapeutics, Faculty of Veterinary Medicine, Damanhour University, Damanhour, 22511 AlBeheira Egypt; 9 University Centre for Research & Development, Chandigarh University, Punjab, India; 10 Department of Research & Development, Funogen, 11741 Athens, Greece; 11 University Hospital Witten-Herdecke, University of Witten-Herdecke, Heusnerstrasse 40, 42283 Wuppertal, Germany; 12 Department of Forensic medicine and clinical toxicology, Faculty of Medicine, Beni-Suef University, 62511 Beni Suef, Egypt; 13 Department of Food Science, Faculty of Agriculture, Zagazig University, Zagazig, 44519 Egypt; 14 School of Food Science and Engineering, South China University of Technology, Guangzhou, 510641 China

**Keywords:** IBD, Ulcerative colitis, Crohn’s disease, *EZH2*, *KRT-14*, Pumpkin seed oil

## Abstract

Inflammatory bowel disease is a collection of intestinal disorders that cause inflammation in the digestive tract. Prolonged inflammation in the gastrointestinal tract is a major risk factor for colorectal cancer. The objective of this study was to fucus on gene expression levels of (*KRT-14*; associated with epithelial cell integrity) and enhancer of zeste homolog-1 (*EZH-2;* involved in cellular proliferation) in a IBD rat model in order to rule out impact of nutraceuticals (pumpkin seed oil; PSO) as a complementary approach to conventional treatments of IBD. In the current study, IBD was induced using dextran sodium sulfate (DSS). Following acclimatization, rats were separated into three groups: the negative control, the positive control, and the treatment group. The DSS (1 ml/kg bw) was given to the positive control and treatment groups. Negative control was given only a normal diet. Pumpkin seed oil (PSO) was given orally as a treatment (0.5 ml/kg bw). Blood and colon tissue were obtained on the 5^th^, 10^th^, 14^th,^ and 18^th^ days. Physical parameters, hematology, biochemical assays, gene expression, and histopathology were carried out. After statistical analyses, macroscopic parameters showed significant differences. Biochemical analyses revealed a significant (*P* ≤ 0.05) decrease in serum potassium concentrations, total cholesterol, triglycerides, total proteins, total oxidants status, and C-reactive proteins in PSO treated group as compared with positive control. Gene expression levels of KRT-14 and EZH2 were significantly (*P* ≤ 0.05) upregulated in PSO treated group as compared to positive control group. Histopathology revealed that pumpkin seed oil preserved the structural integrity of colon.

## Introduction

The intestinal epithelium’s control of its surroundings is applied by tight regulation of its barrier, which is critically positioned between the host immune system and the external environment; both of these can inflict damage. The intestinal barrier controls the bidirectional movement of ions, macromolecules, and water between the host and the lumen, both physically and functionally [1]. A wide range of non-intestinal and intestinal illnesses, including clinically recognized inflammatory bowel disease (IBD), have been linked to intestinal epithelial barrier disruption [2]. The homeostasis of the gastrointestinal tract (GI) is maintained in normal conditions by the limitation of immune reactions against commensal bacteria; nevertheless, in genetically vulnerable individuals, when the limiting control is compromised, the immune response leads to inflammation [3].

Inflammatory bowel diseases (IBD), such as ulcerative colitis (UC) and Crohn’s disease (CD), are chronic conditions that are becoming more common around the world [4]. Because IBD is a chronic, life-long disease that begins at a young age, its prevalence is predicted to rise in the next few decades. IBD mostly affects young individuals and can have serious effects on their quality of life, health care, and work productivity [5]. Despite considerable advancements in therapeutic resources and advances in our understanding of disease pathophysiology, there is still no cure for IBD, and drug-free exemption is not attainable in the majority of cases [6].

A wide spectrum of GI diseases is now treated with pharmaceuticals. Patients with IBD are now focused on using natural substances; thus, herbs have recently emerged as an alternative treatment for inflammatory disorders like IBD. As a result, (CAM) complementary and alternative treatments, particularly herbal remedies, have gotten a lot of scrutiny [3]. Pumpkin (species Cucurbita) is a fruit that grows on the European and American continents, as well as in the Caribbean. Linoleic acid, vitamins E, B, and A, oleic acid, carbohydrates, selenium, phytosterols, and zinc are all abundant in the seeds [7]. Pumpkin (*Cucurbita pepo L*.) is the most widely farmed cucurbit crop in the world [8]. In recent years, pumpkin seed oil has acquired incredible consideration as an eatable oil as well as a potential nutraceutical [9]. Pumpkin seed oil has other beneficial properties like anti-hypertensive, anti-microbial, anti-arthritic, anti-depressive, and anti-inflammatory activities. Other studies have also pointed out the health properties of pumpkin seed oil against neonatal meningitis, diabetes, severe abdominal cramps, and diarrhea [10]. Pumpkin seeds also possess an oleic acid ester of hydroxy oleic acid. This constituent has been shown to decrease cytokine levels to attenuate inflammation in a dose-dependent fashion [11].

Following any tissue injury, a restorative and regenerative program is initiated that aims at the restoration of the function and structure of the injured organ [12]. In recent decades, the recognition of the vital role of epigenetic modifications in modifying the actions of regulatory genes involved in differentiation, tissue renewal, and lineage specification has been raised [13].

Enhancer of zeste homolog 2 (EZH2)-mediated trimethylation of histone 3 lysine 27 is of importance in immune regulation. But there is a lack of evidence to evaluate the effect of EZH2 on regeneration [14]. EZH2 is involved in initiation and maintenance of immune environment which is critical in conditions like IBD [15]. Keratins are involved in the pathogenesis of multiple colorectal disorders, including IBD and cancer. Keratins are known to be critical for the maintenance of epithelium integrity as well as protection against stresses, either nonmechanical or mechanical [16], and to control effects exerted by signaling pathways. Keratins are also involved in cell-death signaling pathways [17]. It was found that keratins are also involved in the regeneration of pneumocytes; however, the mechanism is uncertain. Previous findings suggested that KRT14 is a marker of early proliferation [18].

As EZH-2 is a regulator of the immune system, and in IBD there is disruption of the immune system due to inflammation, the current study aims at exploring the potential of pumpkin seed oil on EZH-2 and to rule out engagement of KRT-14 in epithelial injury and repair in an inflammatory bowel disease rat model.

## Experimental Procedures

### Chemicals and Reagents

Dextran sodium sulfate (DSS) was bought from Sigma Aldrich (United Kingdom) and pure pumpkin seed oil (100% pure) was purchased from Karachi Pansar 120 ml (cold pressed).

### Experimental Design: Animal Grouping and Housing Conditions

Experiments were conducted on *male* Wistar albino rats (N = 48) weighing (150–200 g; age 8–10 weeks). Rats were purchased from the animal breeding facility of the Department of Physiology, Government College University Faisalabad (GCUF). Rats were housed in the experimental animal station of the Department of Physiology, GCUF. A controlled temperature of 25 ± 2 °C was maintained throughout the experiment, and access to food pellets and water was given. Permission from the Ethical Review Committee of GCUF was obtained (Ref# GCUF-ERC-06). Rats (N = 48) were acclimatized to the environmental conditions of the animal house. Following adaptation, rats were equally and randomly divided into three groups. The negative control group (NC; n = 16) was fed normal feed and water. In order to induce IBD, rats (n = 32) were regularly given DSS (1 ml/kg/bw) intra-rectally along with normal chow maintenance diet and water. After confirmation of IBD, rats were divided randomly, into positive control group (PC) and treatment group (PSO; n = 16/group). The treatment group received a daily oral dose of pumpkin seed oil at 0.5 ml/kg/bw as reported by literature [19, 20]. *Pumpkin seed oil is rich in unsaturated fatty acids, antioxidants, and other bioactive compounds. At this dosage, researchers can effectively study its effects on various health parameters without overwhelming the animals’ systems. Oral administration allows nutraceuticals to be processed in a way that can maximize bioavailability and efficacy. Rats in control group were administered with sterile water daily till the end of study period*. The duration of the trial was 18 days; however, decapitation was carried out at different intervals (Days 5^th^, 10^th^, 14^th^ & 18^th^) to monitor the gradual effect of the given treatment on biomarkers. At each decapitation (n = 4), rats were decapitated from each group.

#### Induction of IBD

IBD was induced by using dextran sodium sulfate (DSS, M_w_ > 500,000, 98% pure, Sigma-Aldrich, USA) which is reported to cause inflammation in the colon, although the mechanism is still unclear. However, it is suggested that it causes damage to the epithelial cell layer of the colon, leading to inflammation [21]. The dose of DSS was prepared by dissolving 2.5 g of DSS in 50 ml of distilled water. Single dose of one ml/kgbw was injected intra-rectally for three days in all rats except negative control group and in order to maintain stability of induction throughout study. To evaluate success of IBD model, macroscopic examinations were carried out e.g. diarrhea, physical appearance of colonic end. Moreover, colon weight/length ratio, and colon thickness were also observed as mentioned.

#### Collection of samples

Rats were given mild anesthesia with isoflurane (1–3% concentration), by keeping rats in a closed container and decapitated afterward. Tissues (blood and colon) were acquired for biochemical, gene expression, and histological investigations. Blood samples were taken in two vials. One part of the blood was taken in EDTA tubes for hematology analyses, while the other part was taken in coagulant vials. For serum separation, centrifugation was carried out at 4000×g for 10 minutes. The supernatant was stored at -20 °C for further analysis. For histopathology, one part of the colon tissue was fixed in neutral buffered formalin (10%). The other part was soaked in Trizol for RNA extraction.

### Physical Parameters

#### Body weight

Body weight (g) was documented at the beginning of the experiment. Later on, it was recorded at the following intervals: 5^th^, 10^th^, 14^th^, and 18^th^ days.

#### Macroscopic parameters

The macroscopic examination includes colon weight/length ratio, thickness of the colon, and diarrhea. It was carried out by following the method explained by Periasamy et al. [22]. The distal portion (8 cm) of the colon was taken following decapitation and was rinsed with normal saline afterward. Colon thickness, weight/length ratio, and consistency of stool (diarrhea) were evaluated by the following methods explained by Bobin-Dubigeon et al. [23]. Stool from each rat was scored based on the following scale: 1,normal stool; 2, loose pellet-shaped stool; 3, loose stool, no pellets; and 4, severe diarrhea with mucosa.

### Biochemical Parameters

#### Serum electrolytes

Electrolyte concentrations, including sodium, potassium, and chloride, were analyzed using an Electrolyte Fully Automated AU700 Beckman Coulter.

#### Anti-inflammatory marker (C-reactive protein)

Determination of the C-reactive protein (CRP) in serum was carried out using the commercially available kit from Siemens (REF-DF34).

#### Oxidative stress markers

##### Total antioxidant capacity (TAC; mmol Trolox _equiv_./L)

The procedure for determining TAC in serum samples was previously defined by Rasul et al. [24]. In short, the presence of antioxidants in the sample results in the bleaching of the orthodianisidine color in the assay reagent. Trolox was used as standard to measure the total antioxidant capacity. The higher the antioxidant concentration is present, the more bleaching will occur, which will result in reduced absorbance of samples. The biochromatic wavelength (660 and 870 nm) was calibrated using Trolox standards at 1, 3, 5, and 7 mmol/L concentrations on a semi-auto analyzer by the Biolab 310®. The lowest observable value of 0.19 mmol/7 and linearity of up to 8 mmol Troloxequivalent/l are seen with this assay, with a coefficient of variance < 10% and up to 8 mmol Troloxequivalent/l, with a coefficient of variance [25].

##### Total oxidant status (TOS; μmol _H2O2 equiv._/L)

The concentration of TOS in serum samples was determined by a method previously used by Nisar et al. [26]. The standard curve was constructed from different hydrogen peroxide (H_2_O_2_) concentrations used as standard, and the TOS was represented as μmol H_2_O_2_ equivalent/l. The assay’s detection range was < 10%, and linearity was up to 250 μMol of H_2_O_2_ equivalent /l [27].

##### Serum malondialdehyde (MDA; μmol/L)

Concentration of MDA in serum was carried out by using the thiobarbituric reactive substance assay (TBARS) [28], which relies on thiobarbituric acid, which gives off the pink color-producing reaction of MDA. Absorbance was taken at 532 nm by using a semi-automatic analyzer (Biolab-310, Biobase, Jinan, China) against a blank taken as distilled water.

#### Profile of lipids

Crescent Diagnostic System kits were used for the estimation of total cholesterol (Cat No. CS. 603), high-density lipoprotein cholesterol (Cat No. CS. 606), and triglycerides (Cat No. CS. 611). The instructions mentioned in the manuals of kits were followed for the determination of serum total cholesterol, serum triglycerides, and serum HDL-cholesterol level. The Friedrick equation was used for the determination of low-density lipoprotein concentration [25].

#### Liver function markers

##### Serum liver enzymes

Systemic inflammation by IBD might affect liver, thus liver markers can indicate liver stress or damage. To assess liver enzyme profile, commercially available kits (LAB KIT; Barcelona, Spain) were used for determining the serum Aspartate transaminase (AST) (REF-30243) and serum Alanine transaminase (ALT) (REF-30253) levels. The methods explained in the manuals were followed, and absorbance was taken using an automated biochemical analyzer.

##### Total protein, albumin, and bilirubin (g/dl)

Commercially available kits (DELMA) were used for the determination of total protein (REF-D-638001), albumin (REF-D-601001), and bilirubin (REF-BIL099100 Biomed Diagnostics) levels in serum samples. The manufacturer’s instructions were followed for the assay procedure. The concentration of serum globulin in the sample was calculated by subtracting the values of serum albumin levels from total protein levels.

#### Hematological analysis

Blood samples were collected in an ETDA tube that was used for hematological analysis. A semi-automatic hematology analyzer (Norma Icon-3 Semi-automated 3-part differential hematology system by Norma Diagnostics) was used to measure the complete blood count, including white blood cells, red blood cells, platelets, hemoglobin, and hematocrit.

#### RNA isolation and qRT-PCR

To perform RNA isolation from tissue samples of the colon, the Trizole (ThermoFisher Scientific, Massachusetts, USA) method was used. After isolation, RNA samples were quantified using nanodrops. DNA was synthesized using a Revert Aid cDNA synthesis kit (ThermoFisher Scientific). The manufacturer’s instructions were followed, and equal concentrations of RNA from each sample were used for synthesis. Maxima SYBR Green/ROX Master Mix (ThermoFisher Scientific) was used to perform quantitative real-time PCR (qRT-PCR) on an i*Q5 Bio-Rad* machine. The mRNA expression levels of the EZH-2 and KRT-14 genes were determined by using glyceraldehyde-3-phosphate dehydrogenase as the housekeeping gene. Primers were supplied by Macrogen, South Korea. Primer sequences for both genes are presented in Table 1. Finally, the qRT-PCR data was analyzed using the 2*(- Δ Δct) method.

**Table 1:** Name and sequence of primers

Genes	Sequence
*EZH2-F*	CTTCTCACCAGCTGCAAAGTG
*EZH2-R*	GAGTTGTGTTTTCCCACTGGA
*Krt-14-F*	TACGGGGGCATGTCTCGTTTC
*Krt-14-R*	GTGGCCAAGCGGTCATTGAG

#### Histopathology protocol

Colon tissues were soaked in 10% neutral buffered formalin, and after a series of dehydrations, tissues were embedded in paraffin to make the blocks. Slicing of 4 to 5 μm was carried out with a microtome [Bk-Mt268m; Biobase Biodustry (Shandong) Co., Ltd.]. The tissue sections were placed on a glass slide by using albumin as a coating medium. Tissue samples went through deparaffinization, rehydration, and finally staining with hematoxylin and eosin stain. After staining, mounting was carried out using DPX. Then slides were examined under the microscope, and image quantification was performed by Image J software.

### Statistical Analysis

Two-way analyses of variance (ANOVA) (treatment x time) was used to rule out the statistical significance of the data [27] by setting the *P* ≤ 0.05. Graphpad Prism 8.4 was employed. Costat version 2 was used to perform a post-significant test of the Dunacun Multiple Range Test as a post-hoc significant test [29]. The data is represented as the mean ± SEM.

## Results

### Physical Parameters

#### Body weight (gram)

**Fig. 1:**
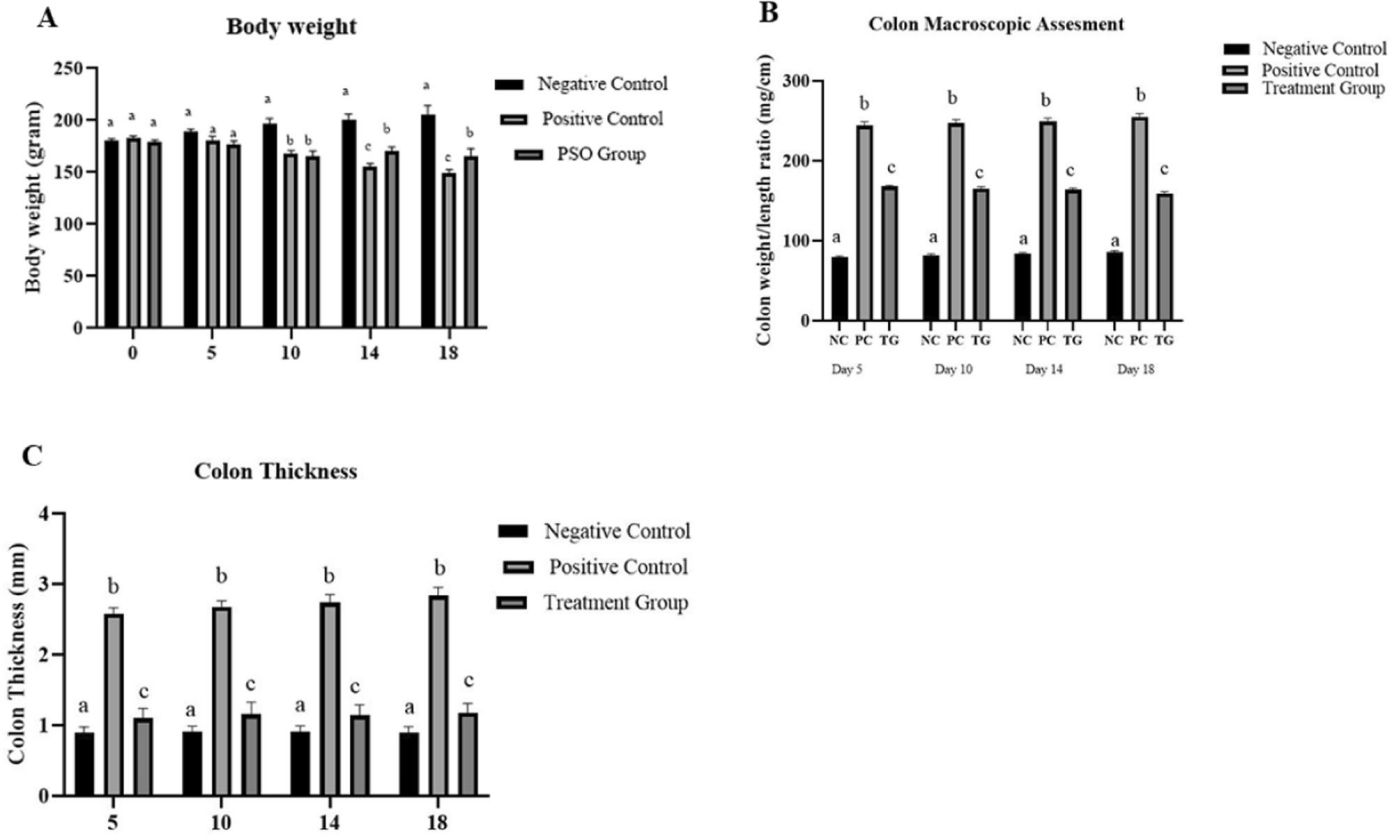
Effect of pumpkin seed oil on physical parameters. **A** Body Weight (gram). **B** Colon thickness. **C** Colon weight/length ratio (g/cm). Normal Control (NC), Positive Control (PC), and pumpkin seed oil treated group at different days in dextran sodium sulfate (DSS) induced IBD rat model. Bars are displayed on basis of Mean ± SE values. Various superscripts are showing significant difference at *P* ≤ 0.05

The analysis of the overall mean body weight of rats showed that at days 0 and 5, there was a non-significant difference between all groups. However, there was a significant decline (*p* ≤ 0.05) in the mean body weight of the positive control group at day 10 (175 ± 0.10), day 14 (159 ± 0.15), and day 18 (148 ± 0.16) as compared to PSO treated group where pumpkin seed oil protected against rapid decline in body weight after IBD induction (Fig. 1).

#### Macroscopy

Macroscopic examination of the colon was done at days 5, 10, 14, and 18; however, the percentage of diarrhea was monitored at day 18. Analyses revealed that there was a significant (*P* ≤ 0.05) increase in the colon weight/length ratio and colon thickness in the positive control group as compared to PSO treated group. Colon thickness consistently increased from 2 mm to 3 mm (day 5 to day 18) in the positive control group along with weight/length ratio from 205 ± 2.0 to 235 ± 3.4 mg/cm (day 5 to day 18) as compared to PSO treated group, where pumpkin seed oil significantly improved these alterations in the treatment group (Fig. 1).

**Fig. 2:**
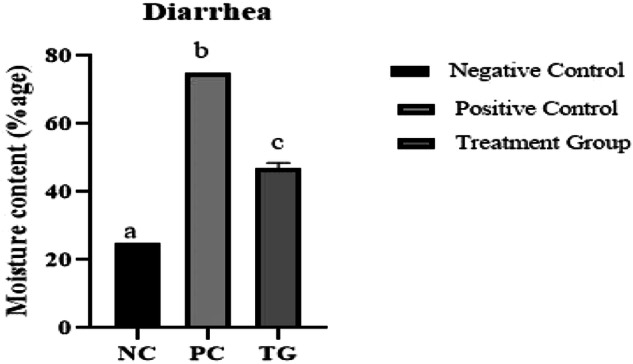
Effect of pumpkin seed oil on Diarrhea (%age) in Normal Control (NC), Positive Control (PC), and pumpkin seed oil treated group at different days in dextran sodium sulfate (DSS) induced IBD rat model. Bars are displayed on basis of Mean ± SE values. Various superscripts are showing significant difference at *P* ≤ 0.05

Similarly, the percentage of diarrhea was significantly high in the positive control group (≥75%) following induction as compared to the PSO treated group where it was rectified (<50%) as shown in Fig. 2.

### Biochemical Parameters

#### Serum electrolytes

**Fig. 3:**
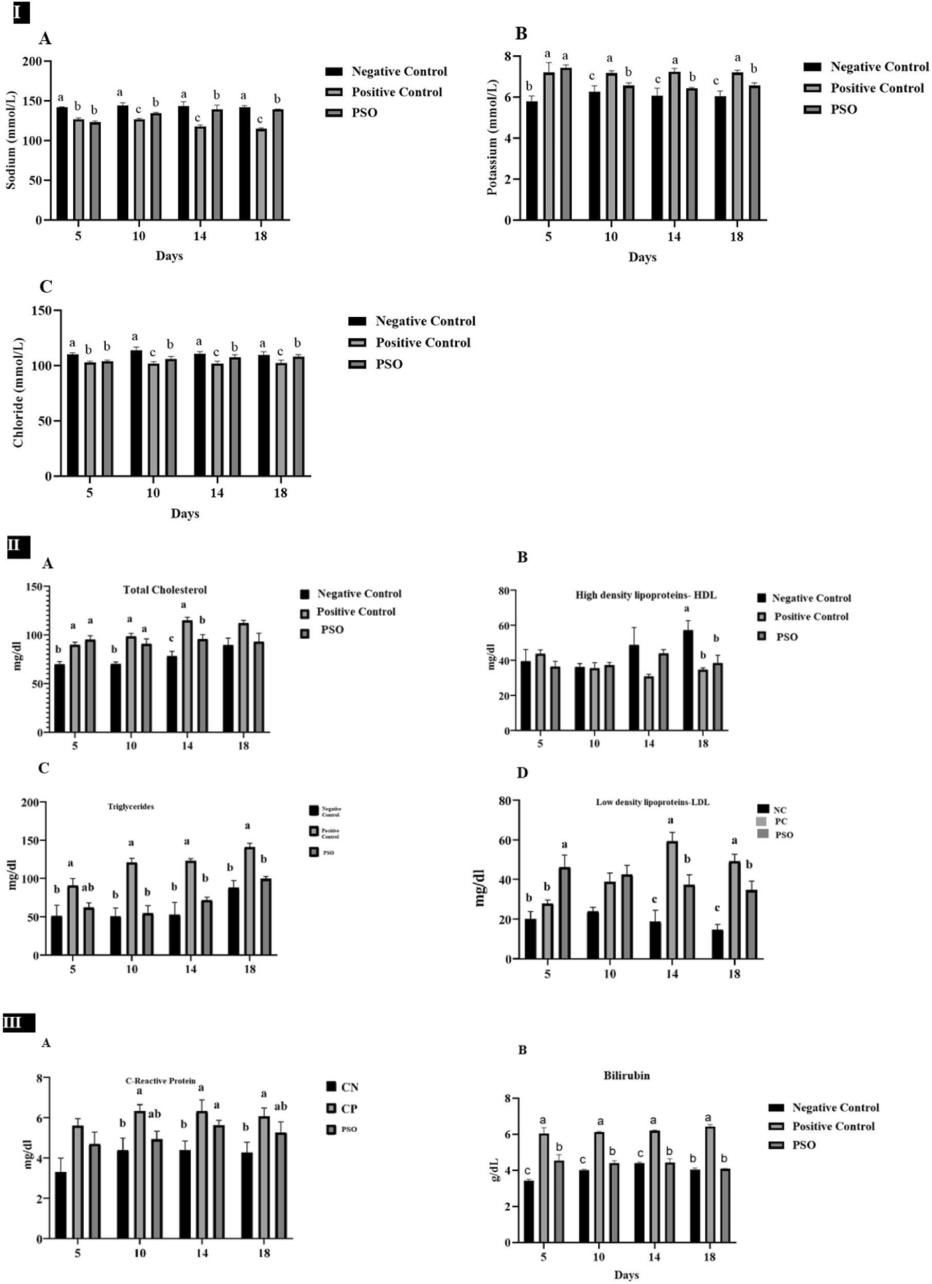
**I**: Effect of pumpkin seed oil on serum electrolytes (**A** Sodium, **B** Chloride, **C** Potassium) (mmol/L). **II**: Effect of pumpkin seed oil on serum lipid profile (**A** Total cholesterol (TC), **B** Triglyceride (TG), **C** High-density lipoprotein (HDL), **D** Low-density lipoprotein (LDL) (mg/dl). **III**: Effect of pumpkin seed oil on serum biochemical parameters (**A** C-reactive protein (CRP) (mg/dl), **B** Serum total bilirubin (g/dl)). Normal Control (NC), Positive Control (PC), and pumpkin seed oil treated group at different days in dextran sodium sulfate (DSS) induced IBD rat model. Bars are displayed on basis of Mean ± SE values. Various superscripts are showing significant difference at *P* ≤ 0.05

After statistical analysis, the overall mean values of serum sodium, potassium, and chloride revealed that there was a significant difference (*P* ≤ 0.05) among all groups at all intervals. The serum concentrations of sodium and chloride showed a progressive decline with concomitant increase in potassium levels in the positive control group as compared to PSO treated group as shown in Fig. 3.

#### Serum Anti-Inflammatory Parameters: C-Reactive Protein

Serum C-reactive protein results showed a significant difference (*P* ≤ 0.05) among all groups at days 10, 14, and 18. The level of C-reactive protein was statistically increased (*P* ≤ 0.05) in the positive control group as compared to the negative control group and PSO treatment group (Fig. 3).

#### Oxidative stress markers

**Fig. 4:**
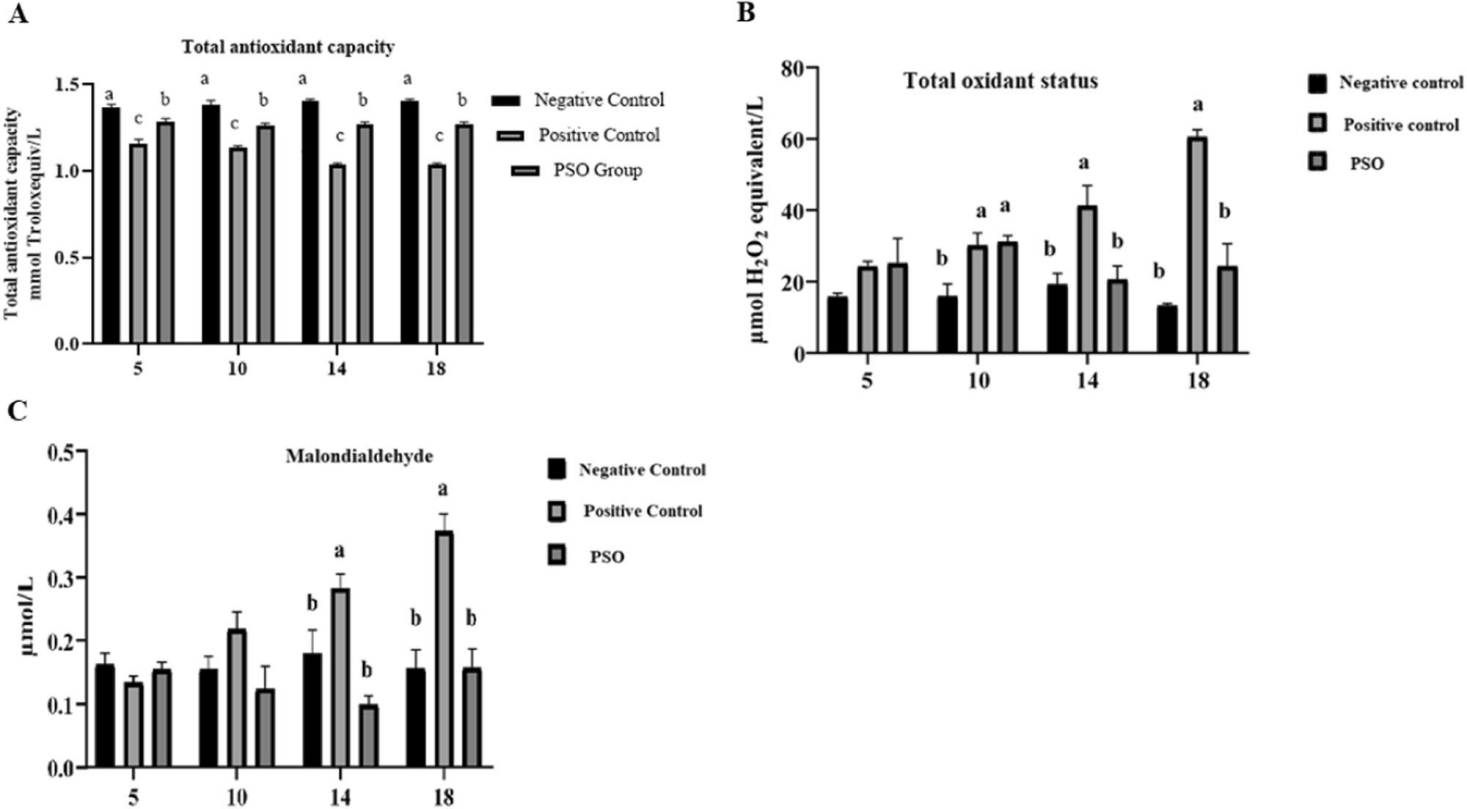
Effect of pumpkin seed oil on serum markers of oxidative stress (Total antioxidant capacity (TAC) mmol Trolox equivalent/L, Total oxidative stress (TOS) µmol H_2_O_2_ equivalent/L, Malondialdehyde (MDA) µmol/L in Normal Control (NC), Positive Control (PC), and Pumpkin seed oil treated group at different days in dextran sodium sulfate (DSS) induced IBD rat model. Bars are displayed on basis of Mean ± SE values. Various superscripts are showing significant difference at *P* ≤ 0.05

The level of total antioxidant capacity (TAC) was significantly increased (*P* ≤ 0.05) in the PSO treated group as compared to the positive control group and negative control group at all days. However, levels of total oxidant status (TOS) and malondialdehyde (MDA) were significantly (*P* ≤ 0.05) increased in the positive control group as compared to PSO treated group. No significant difference in TOS was observed at day 5 and in MDA at days 5 and 10 (Fig. 4).

#### Serum lipid profile

Lipid profile results showed the levels of total cholesterol, triglycerides, and low-density lipoproteins were significantly (*P* ≤ 0.05) higher in the positive control group as compared to the negative control group and PSO treated group (Fig. 3).

#### Liver function markers

The levels of aspartate aminotransferase (AST) and Alanine Transferase (ALT) were significantly increased in the positive control group as compared to the negative control group and PSO treated group. On different days, a significant difference was observed in ALT levels as shown in Table 2.

**Table 2:** Liver function parameters

Aspartate Aminotransferase (AST) levels (IU/L)					
Groups	DAY 5	DAY 10	DAY 14	DAY 18	Overall mean
Control negative	95.56 ± 8.17	98.7 ± 2.66^b^	103.71 ± 7.23	104.125 ± 12.25	100.52 ± 2.06^B^
Control positive	126.49 ± 4.05	119.93 ± 4.72^ab^	124.83 ± 8.65	134.75 ± 7.79	126.5 ± 3.08^A^
Treatment	116.33 ± 7.53	111.33 ± 2.33^a^	112.66 ± 3.38	117.33 ± 6.66	114.41 ± 1.43^A^
Overall mean	112.79 ± 9.10	109.98 ± 6.16	113.73 ± 6.12	118.73 ± 8.86	
Alanine Amino Transferase (ALT) levels (IU/L)					
Control negative	26.13 ± 7.02^b^	29.10 ± 8.53^c^	34.85 ± 5.86^c^	32.26 ± 1.71^c^	30.58 ± 1.89^C^
Control positive	122.61 ± 3.04^a^	110.27 ± 9.89^a^	129.41 ± 11.61^a^	140.61 ± 5.00^a^	125.72 ± 6.34^A^
Treatment	113.66 ± 14.26^a^	64 ± 3.46^b^	82.66 ± 6.38^b^	95.33 ± 7.31^b^	88.91 ± 10.46^B^
Overall mean	87.46 ± 30.77 ^A^	67.79 ± 23.50^A^	82.30 ± 27.29^A^	89.4 ± 31.41^B^	
Total Protein levels (g/dl)					
Control negative	31.24 ± 1.15^b^	29.67 ± 1.48^b^	28.35 ± 1.06^b^	26.77 ± 2.177	29.00 ± 0.95^C^
Control positive	35.66 ± 0.70^a^	36.93 ± 1.91^a^	41.30 ± 0.73^a^	35.98 ± 2.36	37.46 ± 1.30^A^
Treatment	32.44 ± 2.34^a^	33.88 ± 1.57^a^	36.93 ± 1.90^a^	33.36 ± 1.23	34.15 ± 0.97^B^
Overall mean	33.11 ± 1.31^A^	33.49 ± 2.10^A^	35.52 ± 3.80^AB^	32.03 ± 2.73^B^	
Albumin levels (g/dl)					
Control negative	25.14 ± 1.56^a^	24.11 ± 0.87^a^	24.54 ± 2.65	21.47 ± 2.01	23.81 ± 0.80
Control positive	21.86 ± 0.78^b^	22.9 ± 0.62^b^	23.04 ± 1.41	24.02 ± 0.45	22.95 ± 0.44
Treatment	23.24 ± 1.14^ab^	21.59 ± 0.29^ab^	23.84 ± 2.47	23.00 ± 1.53	22.91 ± 0.47
Overall mean	23.41 ± 0.95	22.86 ± 0.72	23.80 ± 0.43	22.83 ± 0.74	
Globulin levels (g/dl)					
Control negative	4.91 ± 0.27^c^	4.71 ± 0.75^b^	3.80 ± 2.49^b^	4.12 ± 5.64	4.38 ± 1.97^C^
Control positive	13.79 ± 0.80^a^	13.98 ± 1.35^a^	18.26 ± 1.56^a^	11.95 ± 3.86	14.49 ± 0.95^A^
Treatment	9.20 ± 1.19^b^	12.29 ± 1.86^a^	13.08 ± 4.20^ab^	10.35 ± 2.73	11.23 ± 0.88^B^
Overall mean	9.3 ± 4.01	10.32 ± 3.38	11.71 ± 4.22	8.80 ± 2.20	

Aspartate Aminotransferase level (IU/L; mean ± S.E in (NC); Alanine Amino Transferase level (IU/L; mean ± S.E); Total Protein level (g/dl; mean ± S.E); Albumin level (g/dl; mean ± S.E); and Globulin level (g/dl; mean ± S.E) in negative control, (PC) positive control and (PSO) pumpkin seed oil treated group at different days in dextran sodium sulfate induced IBD rat model. ^A, B^Mean having different letter are significantly different from each other (*P* ≤ 0.05) ^a, b^Mean having different letter are significantly different from each other (*P* ≤ 0.05).

The result also exhibited a significant increase (*P* ≤ 0.05) in total protein (g/dl), globulin (g/dl), and bilirubin (g/dl) in the positive control group as compared to the negative control group and PSO treated group, as shown in Table 2. There was no significant difference in the level of albumin (g/dl) among all groups as shown in Table 2.

### Hematologic Parameters

There was a significant (*P* ≤ 0.05) increase in the levels of white blood cells and hemoglobin levels in positive control group as compared to PSO treated group. However, levels of red blood cells, haematocrit, and platelets were statistically non-significant (*P* ≥ 0.05) among all groups as shown in Table 3.

**Table 3:** Hematological parameters

White blood cells level (10^3^/µl)					
Groups	DAY 5	DAY 10	DAY 14	DAY 18	Overall mean
Control negative	11.7 ± 0.45	10.1 ± 1.17^b^	7.07 ± 0.45^b^	12.5 ± 0.15	10.34 ± 1.19^b^
Control positive	12.43 ± 0.92	17.83 ± 1.66^a^	13.26 ± 0.48^a^	12.2 ± 1.11	13.94 ± 1.31^a^
Treatment	13.13 ± 1.15	16.01 ± 1.65^a^	7.21 ± 1.03^b^	13.81 ± 1.93	12.54 ± 1.87^a^
Overall mean	12.42 ± 0.41^a^	14.64 ± 2.33^ab^	9.18 ± 2.04^b^	12.83 ± 0.49^c^	
Red blood cells level (10^6^/µl)					
Control negative	7.05 ± 0.34	7 ± 0.31	7.26 ± 0.30	7.22 ± 0.37	7.13 ± 0.06
Control positive	6.56 ± 0.00	7.51 ± 0.06	7.12 ± 0.24	7.25 ± 0.13	7.11 ± 0.20
Treatment	7.42 ± 0.49	6.45 ± 0.44	7.06 ± 0.23	7.26 ± 0.53	7.04 ± 0.21
Overall mean	7.01 ± 0.24	6.98 ± 0.30	7.14 ± 0.05	7.24 ± 0.01	
Platelets level (10^3^/µl)					
Control negative	1021.33 ± 80.09	1007.33 ± 106.42	767.66 ± 29.86^b^	1020.66 ± 112.94	954.24 ± 62.27
Control positive	674.66 ± 132.47	963.33 ± 122.41	957.66 ± 46.42^a^	1189 ± 188.47	946.16 ± 105.32
Treatment	822.33 ± 89.03	866.33 ± 164.07	878.66 ± 56.93^ab^	804 ± 99.85	842.83 ± 17.71
Overall mean	839.44 ± 100.44	945.66 ± 41.65	867.99 ± 55.10	1004.55 ± 111.43	
Hemoglobin level (g/dl)					
Control negative	13 ± 0.73	12.86 ± 0.52	13.06 ± 0.75^ab^	14.26 ± 0.53	13.29 ± 0.32^ab^
Control positive	13.06 ± 0.61	14.56 ± 0.53	13.93 ± 0.44^a^	14.1 ± 0.64	13.91 ± 0.31^a^
Treatment	13.8 ± 0.75	12.03 ± 1.02	11.46 ± 0.48^b^	12.64 ± 0.73	12.48 ± 0.50^b^
Overall mean	13.28 ± 0.25	13.15 ± 0.74	12.81 ± 0.72	13.66 ± 0.51	
Hematocrit level (%age)					
Control negative	41.13 ± 2.31	39.63 ± 3.86	41.03 ± 0.57^ab^	41.4 ± 1.60	40.79 ± 0.39
Control positive	44.4 ± 0.47	44.1 ± 1.23	42.4 ± 1.47^a^	40.73 ± 0.88	42.90 ± 0.84
Treatment	44.56 ± 2.22	40.03 ± 3.14	38.4 ± 1.02^b^	41.9 ± 3.79	41.22 ± 1.32
Overall mean	43.36 ± 0.64	41.25 ± 2.08	40.61 ± 2.00	41.34 ± 0.58	

White blood cells (10^3^/µl; mean ± S.E); Red blood cells (10^6^/µl; mean ± S.E); Platelet (10^3^/µl; mean ± S.E); Hemoglobin level (g/dl; mean ± S.E); and Hematocrit level (%age; mean ± S.E) in (NC) negative control, (PC) positive control and (PSO) *pumpkin seed oil* treated group at different days in dextran sodium sulfate induced IBD rat model. ^A–C^Means having different letter are significantly different from each other (*P* ≤ 0.05). ^a,b^Means having different letter are significantly different from each other (*P* ≤ 0.05).

### Gene Expression Analysis

**Fig. 5:**
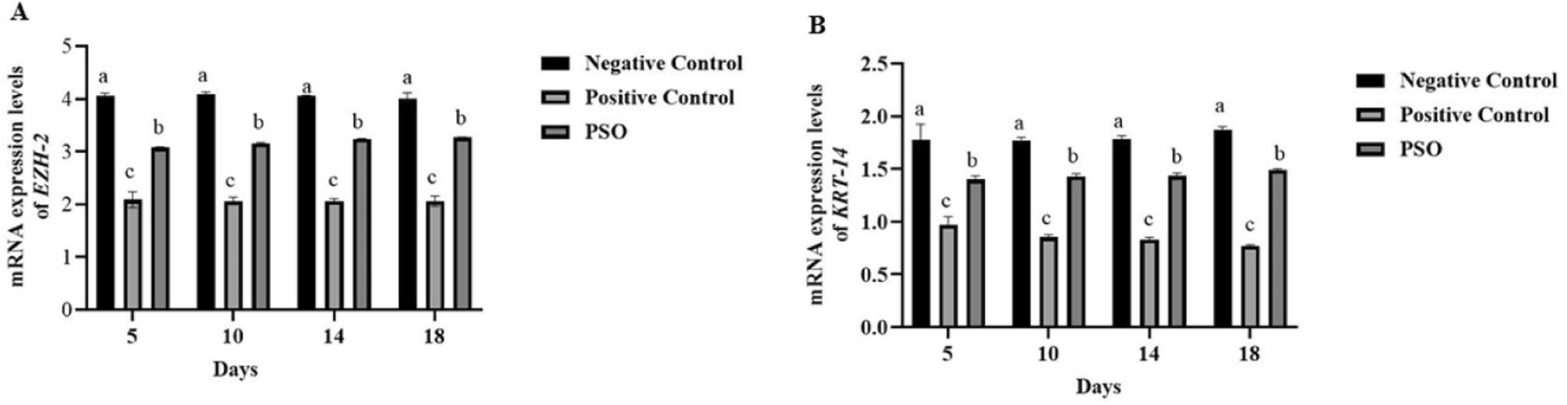
Effect of pumpkin seed oil on gene expression of Enhancer of zeste homolog-2 (*EZH-2*) and Keratin protein-14 (*KRT-14*) in Normal Control (NC), Positive Control (PC), and Pumpkin seed oil treated group at different days in dextran sodium sulfate (DSS) induced IBD rat model labeled as **A** and **B**. Bars are displayed on basis of Mean ± SE values. Various superscripts are showing significant difference at *P* ≤ 0.05

Gene expression analysis revealed a significant downregulation of EZH-2 and KRT-14 levels (*P* ≤ 0.05) in the positive control group at all intervals as compared to negative control and PSO treated group, as observed in Fig. 5.

### Microscopic Examination

On day 5, the negative control group showed normal lumen and mucosa; the positive control group displayed mucosal ulceration, edema and deranged dense, regular connective tissue; and the pumpkin seed oil-treated group showed resettlement in mucosa and lumen.

On day 10, the negative control group revealed the normal architecture of the colon; the positive control group exhibited damage to the mucosa and muscularis propria; and (C) the pumpkin seed oil-treated group showed retrieval of colonic integrity.

On day 14, the negative control group showed typical integrity of the intestinal epithelium; the positive control group revealed impaired mucosa and ulceration; and the pumpkin seed oil-treated group exhibited recuperation of the epithelium.

**Fig. 6:**
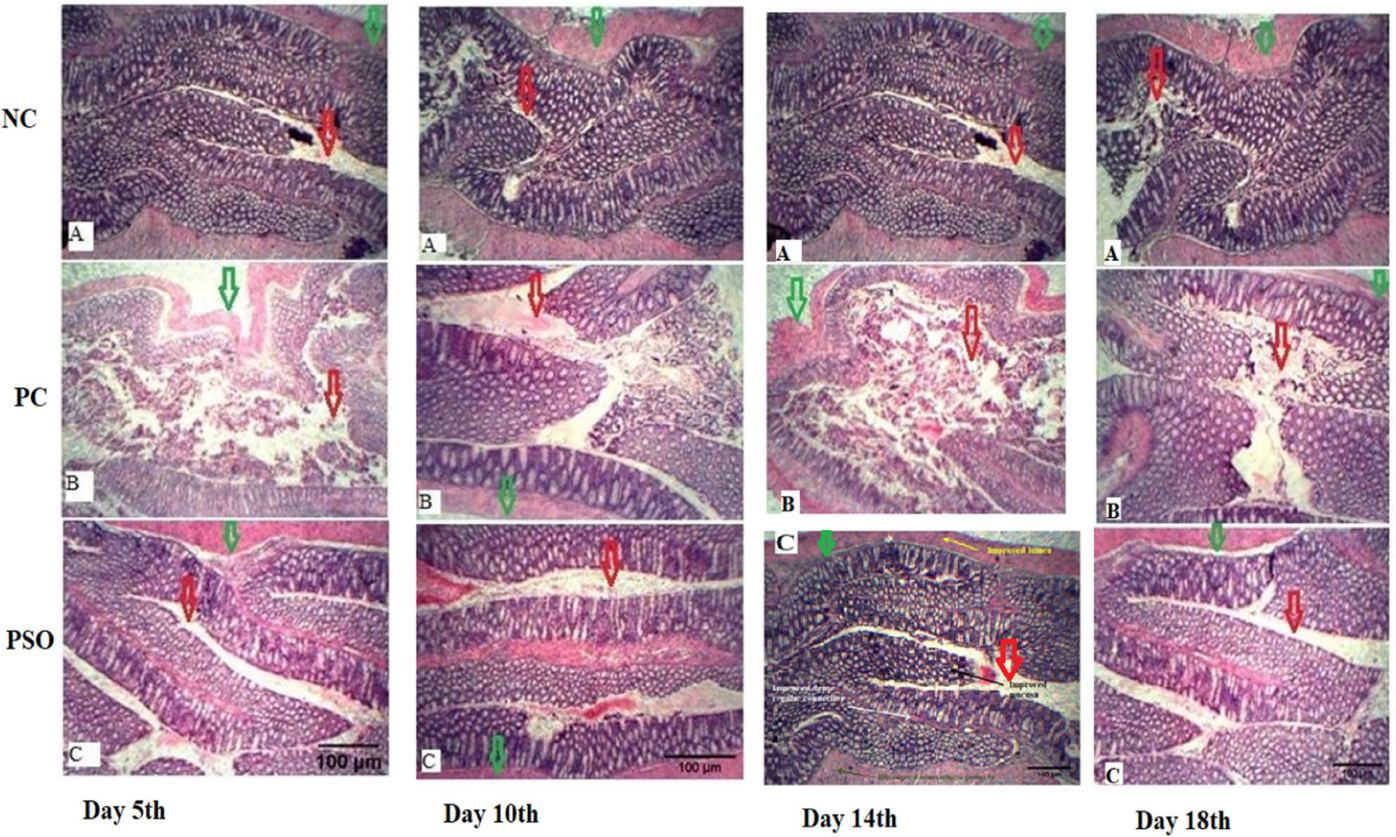
Histological variations by Pumpkin seed oil in rat colon tissue stained with Hematoxylin and Eosin (10X) at day 5 in Dextran Sodium Sulfate (DSS) induced intestinal injury rat model. **I**: Histological examination at day 5: **A** Negative Control (NC) showing normal lumen and mucosa, **B** Positive Control (PC) displaying mucosal ulceration and deranged dense regular connective tissue and **C** Pumpkin seed oil treated group showing resettlement in mucosa and lumen. Scale bar = 100 µm. **II**: Histological examination at day 10: **A** Negative Control (NC) revealing normal architecture of colon, **B** Positive Control (PC) exhibiting damage to mucosa and muscularis propria and **C** Pumpkin seed oil treated group showing retrieval of colonic integrity. Scale bar = 100 µm. **III**: Histological examination at day 14: **A** Negative Control (NC) showing typical integrity of intestinal epithelium, **B** Positive Control (PC) is revealing impaired mucosa and ulceration, and **C** Pumpkin seed oil treated group exhibiting recuperation of epithelium. Scale bar = 100 µm. **IV**: Histological examination at day 18: **A** Negative Control (NC) showing usual appearance of intestinal mucosa, **B** Positive Control (PC) revealing disrupted lumen and mucosa and **C** Pumpkin seed oil treated group showing refining of epithelial structures. Scale bar = 100 µm. Green arrows show epithelial damage and red arrows show the interstitial space

On day 18, the negative control group showed the usual appearance of intestinal mucosa; (B) the positive control (PC) revealed disrupted lumen and mucosa; and (C) the pumpkin seed oil-treated group showed refining of epithelial structures (Fig. 6).

## Discussion

Inflammatory bowel disease is characterized by a decrease in body weight because of the inflammatory conditions, even with a sufficient diet. Weight loss in IBD is mainly due to disturbances in glucose and lipid homeostasis. An increase in lipid oxidation and a decrease in glucose oxidation are major contributors. Another reason for net weight loss in IBD is an increase in energy expenditure [30]. In a current study, a significant net weight loss was observed in rats, more pronounced at days 10, 14, and 18, which can be attributed to the longevity of the disease process. On the contrary, in the pumpkin seed oil-treated group, body weight was restored, and the effects of IBD on body weight loss were rectified. It may be attributed to the high vitamin, antioxidant, and mineral content of PSO, which protects the body cells against oxidative stress [31].

Thickening of the bowel wall may take place due to various pathological mechanisms involved in certain conditions, like infectious, inflammatory, or neoplastic conditions [32]. Thickening of the bowel wall has a correlation with inflammatory activity. It also serves as a predictor of acute disease [33]. In the positive control group, a significant increase in colon wall thickness is a strong marker of the acute phase of IBD. Similarly, the colon weight/length ratio was significantly high in the positive control group. It is stated by [34] that in the colitis model, edema and inflammation in the colonic mucosa presumably cause a decrease in the colon length. Inflamed colons shorten in length and become thicker [35]. The pathogenesis of IBD-associated diarrhea is an outcome of mucosal damage caused by persistent inflammation, resulting in dysregulated intestinal ion transport and impaired epithelial barrier function. Aberrant barrier function further contributes to diarrhea via the leak-flux mechanism [36]. The current study revealed the highest diarrhea percentage in the positive control group, which was subsequently normalized in the treatment group. The antidiarrheal activities of pumpkin seed oil may be attributed to their protection against inflammation and gut irritation [37].

In normal physiology, epithelial cells of the intestine express various channels and ion transporters on the basolateral and apical membranes that assist in the coordination and maintenance of dynamic equilibrium between absorption and electrolyte secretion [36]. Once the equilibrium of electrolyte absorption and secretion is disrupted, it results in fluid loss in diarrhea [38].

In the colon of the mammalian gastrointestinal tract, the predominant route for the absorption of Na^+^, Cl^−^ and water is electroneutral NaCl absorption, whereas ENaC-regulated electrogenic Na^+^ absorption is vital for the maintenance of fluid homeostasis in the distal part of the colon. Impaired Na^+^ absorption is a major contributor to the resulting diarrhea [39]. The current study revealed that serum sodium and chloride levels were significantly decreased in the positive control due to excessive loss in diarrhea, whereas an increase in the levels of potassium was observed, which can be linked to the fact that IBD resulted in inflamed mucosa leading to imbalanced absorption and secretion of electrolytes, often resulting in ionic imbalance. Low sodium and chloride absorption, as well as increased secretion of potassium, are the most common and critical abnormalities [40]. Restored levels of electrolytes in the treatment group may imply that pumpkin seed oil has interfered with electrolyte balance. This can be attributed to the effects of pumpkin seed oil on kidneys that regulate electrolyte balance [41].

C-reactive protein (CRP), which is released by hepatocytes upon stimulation of pro-inflammatory cytokines, is associated with inflammatory bowel disease (IBD) [42]. In the current study, CRP levels were non-significant until day 5; however, a significant rise was observed at days 10^th^, 14^th^, and 18^th^ in the positive control group. In the PSO-treated group, CRP levels were redressed. It was stated by [43] that PSO supplementation had a favorable response to CRP levels by decreasing 16% of CRP values. Also, the effects of pumpkin seed oil in decreasing the inflammatory biomarker with concurrent improvements in lipid biomarkers may be due to increased levels of n-3 fatty acids [44].

Many lines of evidence suggest that IBD is linked with the generation of oxidative stress resulting from an imbalance between ROS and antioxidant activity. This imbalance in IBD in the inflamed mucosa ultimately contributes to chronic tissue damage [45]. The current study revealed that levels of total oxidant status (TOS) and malondialdehyde were significantly high in the positive control group, whereas total antioxidant capacity (TAC) levels were low. Low levels of TAC could be attributed to gastrointestinal losses [46]. Likewise, high MDA [47] and TOS levels in IBD are associated with increased inflammation [48]. The current study also showed that pumpkin seed oil is a recognized source of phenolic compounds [49]. Naturally found plant phenolics possess various important groups of compounds with health-promoting activities, such as phenolics, which may work as antioxidants [50]. Also, pumpkin seed oil is rich in vitamin E, which is considered a powerful antioxidant [51]. From the aforementioned results, it can be implied that pumpkin seed oil effectively ameliorated oxidative stress damage in the treatment group.

Colitis also has disconcerting effects on overall metabolic processes, being involved in energy homeostasis such as lipolysis, fatty acid oxidation, lipogenesis, fatty acid uptake, and cholesterol synthesis, which subsequently cause dyslipidemia [52]. Treatment with PSO showed a positive impact on the lipid profile. It significantly reduced the concentrations of total cholesterol, triglycerides, and low-density lipoproteins and raised the levels of high-density lipoproteins in the positive control group. These findings are attributed to the presence of phytoestrogens in PSO, particularly secoisolariciresinol. This phytoestrogen is confirmed to have a lipid-lowering effect [53].

Any injury to the integrity of the intestinal mucosal barrier leads to IBD and increased permeability, which promotes the release of various proinflammatory cytokines that enter the liver via the gut-liver axis and initiate signaling cascades of inflammation that induce liver injury [54]. Elevated levels of ALT and AST are the sensitive biomarkers of liver injury [55]. Meanwhile, liver injury can induce abnormal lipid metabolism [56]. The current study revealed that levels of AST and ALT were significantly higher in the positive control group, indicating liver injury. Similarly, an increase in the levels of total cholesterol was another indication of liver injury and dyslipidemia. Treatment with pumpkin seed oil significantly lowered the levels of liver enzymes, thus promoting hepatoprotection. It was stated by [57] that PSO can reduce hepatocellular disturbances. This hepatoprotective effect of pumpkin seed oil may be due to the radical-scavenging activity of flavonoids and phenolics by inhibiting cytochrome P-450 aromatase [58].

Two important constituents of serum proteins are albumin and globulin, which play an important role in inflammation [59]. An increased level of globulins is speculated to serve as a marker of inflammation and reflective of a collective exposure of different proinflammatory cytokines [60]. It was observed that levels of total protein, globulin, and total bilirubin were significantly raised in the control-positive group. However, after treating rats with pumpkin seed oil, there was a marked reduction in the levels of total protein, globulin, and bilirubin. The hepatoprotective effects of PSO may be attributed to the rich levels of alpha-linolenic acid, omega-3, and 6 fatty acids and fibers present in PSO [61]. No significant effect was observed at levels of albumin.

Hematology analyses showed that the leukocyte count was significantly elevated in the positive control group. Inflammation and injury sites are attributed to this rise in leukocyte count, as getting leukocytes at the site of insult is very significant [62], and in low-grade inflammation, leukocyte count elevates [63]. In the current study, treatment with PSO normalized the levels of leukocytes due to its immunomodulatory activities [41]. Levels of hemoglobin were also significantly raised in the positive control group. Involvement of the liver due to the gut-liver axis, dyslipidemia, and oxidative stress may increase the levels of hemoglobin. One of the explanations for increased hemoglobin levels and liver injury is oxidative stress catalyzed by the accumulation of iron in excess of physiologic requirements [64]. In the treatment group, hemoglobin levels were restored because PSO is rich in antioxidants and plays a great deal as immunomodulatory. Red blood cell, platelet, and hematocrit levels were non-significant.

EZH2, the catalytic subunit of the polycomb repressive complex (PRC2), is responsible for maintaining homeostasis and the integrity of the intestinal barrier during inflammation. EZH2 overexpression in the intestinal epithelium provides more resistance to colitis in mice [65]. In a current study, there was a downexpression of EZH-2 in the positive control group. It can be implied that impaired intestinal epithelium after inflammation resulted from low levels of EZH-2. On the contrary, overexpression was observed following treatment with PSO. As PSO contains many unsaturated fatty acids and phenolics which might have reduced the inflammation and promoted intestinal restoration by overexpressing levels of EZH-2.

In an acute inflammatory condition, if keratin expression is not adequately restored, it may affect the repair of the mucosa, which leads to the pathogenesis of cancer [66]. Keratin is a cytoskeleton protein responsible for maintaining mechanical barriers under normal conditions. Keratin deficiency can lead to intestinal mucosal damage [67]. From the current study, it is evident that expression levels of KRT-14 in the IBD model, positive control group were significantly reduced, whereas it was overexpressed after treatment with PSO. The decreased expression of Krt-14 following colitis may be attributed to an impaired intestinal barrier. Thus, it is implied that treatment with PSO reduced colonic inflammation and promoted the integrity of the colon by overexpression KRT-14 levels.

Microscopic examination of colonic tissue displayed evident disruption and impaired epithelium in the positive control group. Appreciable microscopic lumen variations were seen that progressed from day 5^th^ to day 18^th^ in order of severity. In IBD, among the various histological parameters, distortion of crypts and an elevated number of inflammatory cells in lamina propria are exhibited [68]. Biochemical and histological parameters both indicated in the current study that during inflammatory bowel disease, multiple factors caused impairment in the epithelial barrier, thus disrupting its structural integrity and functional capacity. Pumpkin seed oil was effective enough to restore not only biochemical parameters but also structural integrity, leading to functional restoration of the intestinal epithelium. Pumpkin seed oil may have the potential to treat IBD and colon cancer by inhibiting hyperplastic cells, normalizing antioxidant enzyme levels, and increasing the ratio (colon length/weight).

## Conclusion

It can be concluded that pumpkin seed oil has treatment against inflammatory bowel disease by regulating the expression levels of *EZH-2* and *KRT-14*. Its effectiveness is due to modulation of antioxidants, attenuation of colon mucosal cells, and rectification of histological architecture. Future studies are recommended to decipher the underlying mechanisms involved in colon protection especially through gene/protein expression testing for antioxidants and inflammatory cytokines.

## Data Availability

Data is available with the corresponding author upon reasonable request.
